# Supplementary material for: Mosquito salivary apyrase regulates blood meal hemostasis and facilitates malaria parasite transmission
Source: Nat Commun. 2024 Sep 18;15:8194. doi: 10.1038/s41467-024-52502-3 (PMC11410810; doi:10.1038/s41467-024-52502-3)
Supplement: Supplementary file 1 — Supplementary Information [file 41467_2024_52502_MOESM1_ESM.pdf]

Supplementary figures

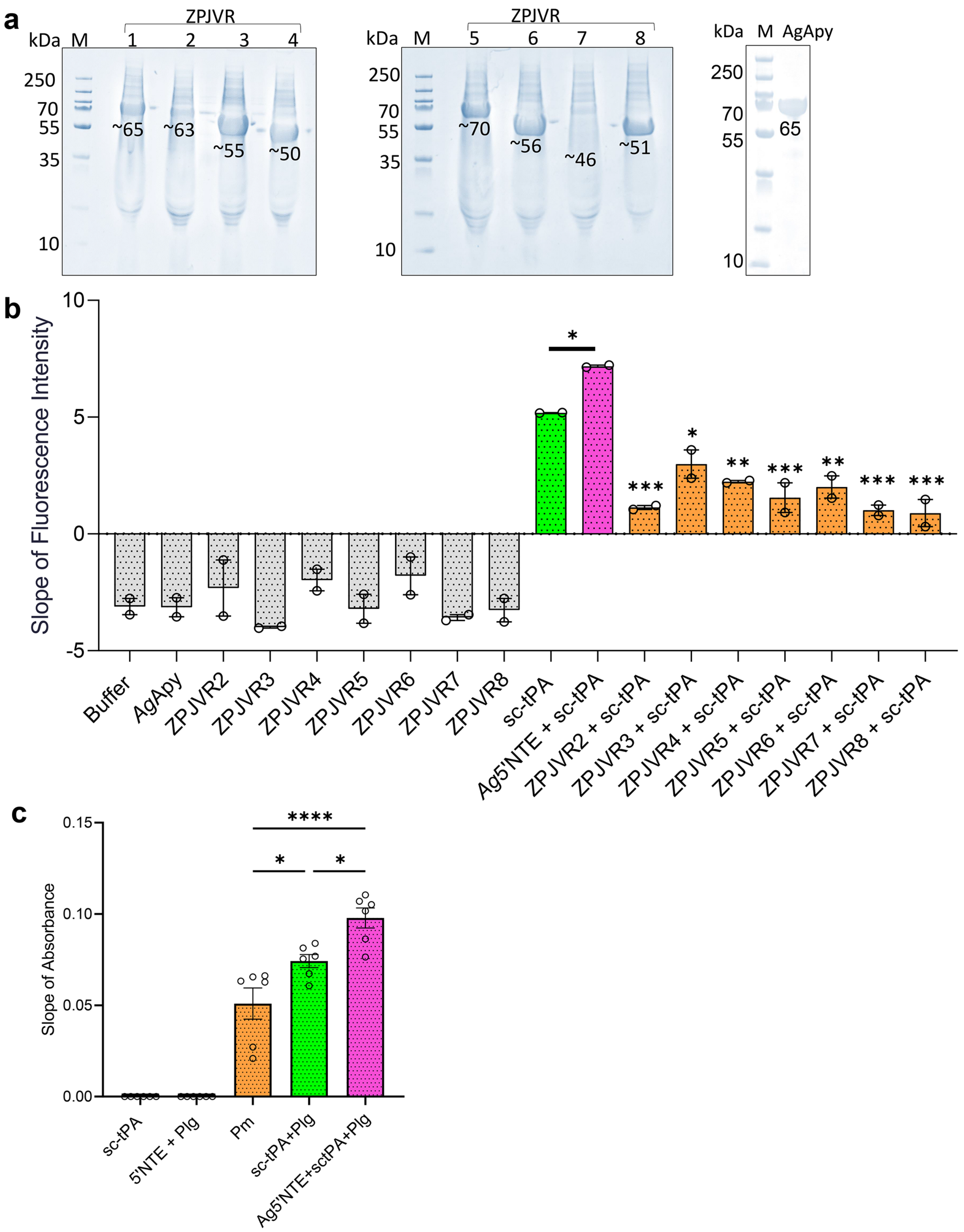

Figure S1: Recombinant protein production and tPA activation assay, related to Figure 1. (**a**) Coomassie stained gel of recombinant proteins for the eight tPA activator candidates. ZPJVR1-8: lab ID for eight candidate proteins; Western blot for AgApyrase using anti-His antibody. M: molecular marker. (**b**) tPA activation fluorogenic assay for the eight identified proteins. All the eight candidate proteins (ZPJVR1-8) were tested for tPA activation and only rAg5'NTE (ZPJVR1, pink bar) activated tPA. Data from two independent experiments. Groups were compared with an ordinary one-way ANOVA followed by Šídák's multiple comparisons test. ***P <0.0001; **P <0.001; *P<0.02; ns: not significant. (**c**) Plasminogen activation in the presence of rAg5' NTE and tPA measured by a colorimetric assay. Ag5'NTE activated tPA in-turn activates plasminogen to plasmin (dark pink) at higher levels than plasmin, sc-TPA or tc-tPA alone. One-way ANOVA with Šídák's multiple comparisons test, *P ≤0.02; ****P <0.0001.

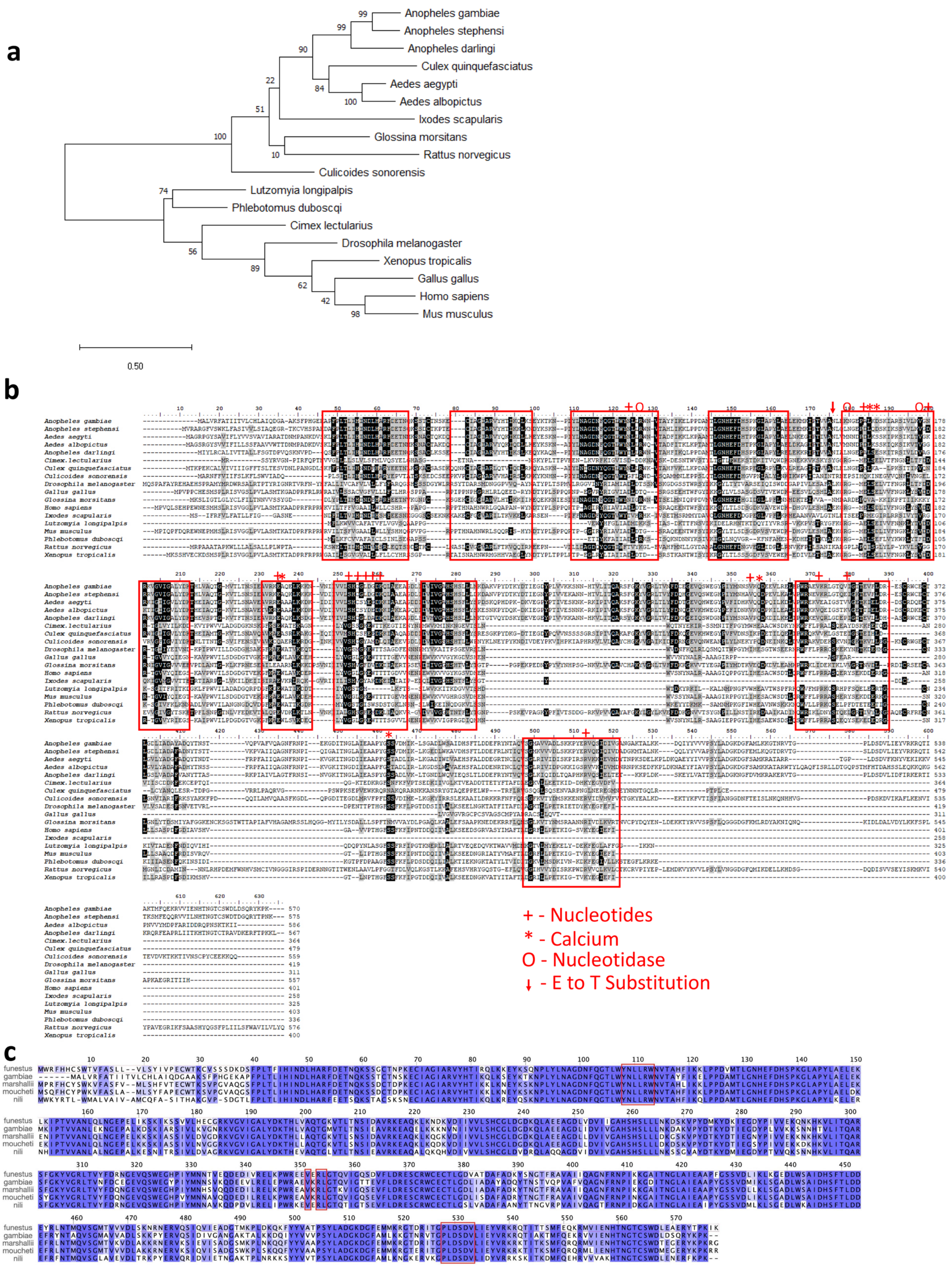
a
Anopheles gambiae
Anopheles stephensi
Anopheles darlingi
Culex quinquefasciatus
Aedes aegypti
Aedes albopictus
Ixodes scapularis
Glossina morsitans
Rattus norvegicus
Culicoides sonorensis
Lutzomyia longipalpis
Phlebotomus duboscqi
Cimex lectularius
Drosophila melanogaster
Xenopus tropicalis
Gallus gallus
Homo sapiens
Mus musculus
0.50
b
+ - Nucleotides
* - Calcium
O - Nucleotidase
↓ - E to T Substitution
c
funestus
gambiae
marshallii
moucheti
nili

Figure S2: Phylogeny of apyrase protein sequences and predicted interaction of AgApyrase with human tPA, related to Figure 2. (**a**) Boot strapping done for 1000 iteration and percentage of trees in which the associated taxa clustered together shown next to the branches. (**b**) Multiple sequence alignment of apyrase sequences. The red boxes depict the conserved region amongst all the species. '+' highlights the residues where nucleotides bind; '*' points the calcium binding site; O points the nucleotidase binding site; ↓ depicts the E to T amino acid substitution in AgApyrase. (**c**) Sequence alignment of apyrases from *An. gambiae*, *An. funestus*, *An. marshallii*, *An. moucheti*, and *An. nili* showing the apyrase residues (highlighted in red boxes) mediating the contact with human tPA. The sequence color indicates the degree of identity.

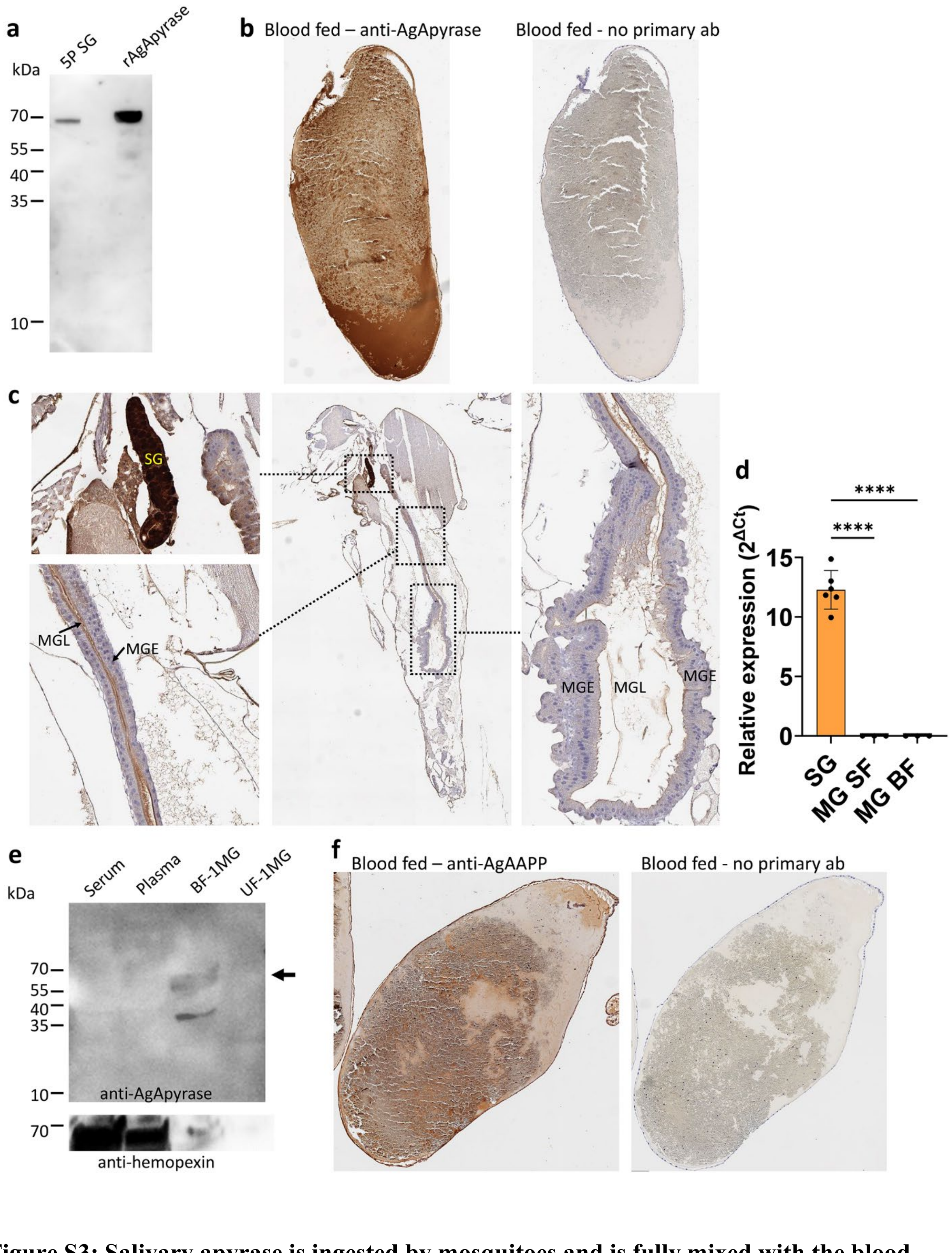

Figure S3: Salivary apyrase is ingested by mosquitoes and is fully mixed with the blood meal, related to Figure 3. (**a**) Western blot showing the reactivity of anti-AgApyrase antibodies

against *An. gambiae* salivary gland protein extracts (5P SG = 5 pairs of salivary glands) or against rAgApyrase protein. Only one band is detected in the western blot showing the specificity of the antibody. (**b**) Immunohistochemistry performed on human blood fed mosquito midgut shows ingestion of salivary AgApyrase which is mixed with the blood bolus content (left panel, dark brown staining). Right panel shows the staining performed on a different section of the same blood fed mosquito midgut and stained only with secondary antibody. (**c**) Immunohistochemistry performed on unfed whole mosquito with anti-apyrase antibodies showed faint brown staining in the foregut lumen and midgut lumen and strong brown staining in the salivary gland (SG). MGE: midgut epithelium; MGL: midgut lumen. (**d**) qPCR analysis showing AgApyrase expression in mosquito salivary glands but not in midguts. Salivary glands (SG) were isolated from sugar-fed mosquitoes whereas midguts were isolated from either sugar-fed (MG SF) or blood-fed (MG BF) mosquitoes. AgApyrase expression was measured relative to the mosquito S7 ribosomal gene. Statistical analyses were performed using an unpaired T-test (GraphPad, San Diego, CA, USA). Each experiment included three biological replicates per condition for midguts and six for salivary gland extracts. One-way ANOVA with Dunnett's multiple comparison, ****$P<0.0001$. (**e**) Western blot analysis showing the specificity of anti-AgApyrase antibodies. The human serum and plasma were tested for the presence of apyrase. No signal was obtained with anti-apyrase antibodies for human serum and plasma and for unfed mosquito midgut. An antibody against human hemopexin was used as loading control for serum and plasma samples. Desired band ~65kDa (arrow) was obtained in blood-fed midgut sample. M: Marker, BF: Blood fed; UF: Unfed; MG: Midgut. (**f**) Immunohistochemistry performed on human blood fed mosquito midgut shows ingestion of another salivary protein, AgAAPP, which is mixed with the blood bolus content.

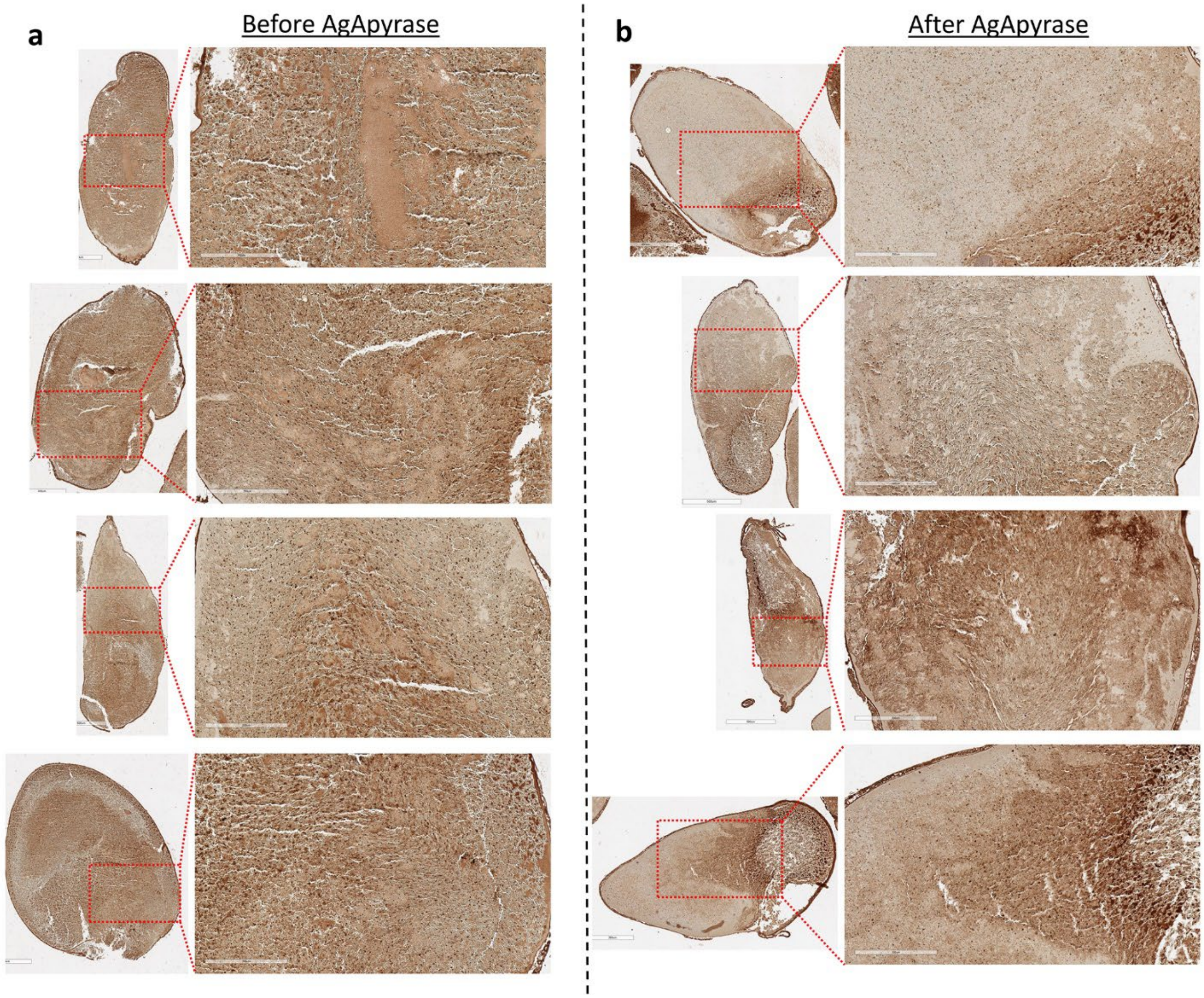

Figure S4: Detection of fibrinogen and fibrin in the mosquito blood bolus, related to Figure 3. Immunohistochemistry of mosquito blood boluses before (a) and after (b) supplementation with rAgApyrase showing fibrin and fibrinogen by staining with an anti-fibrinogen antibody (dark brown staining). A decrease in staining for the after treatment is observed.

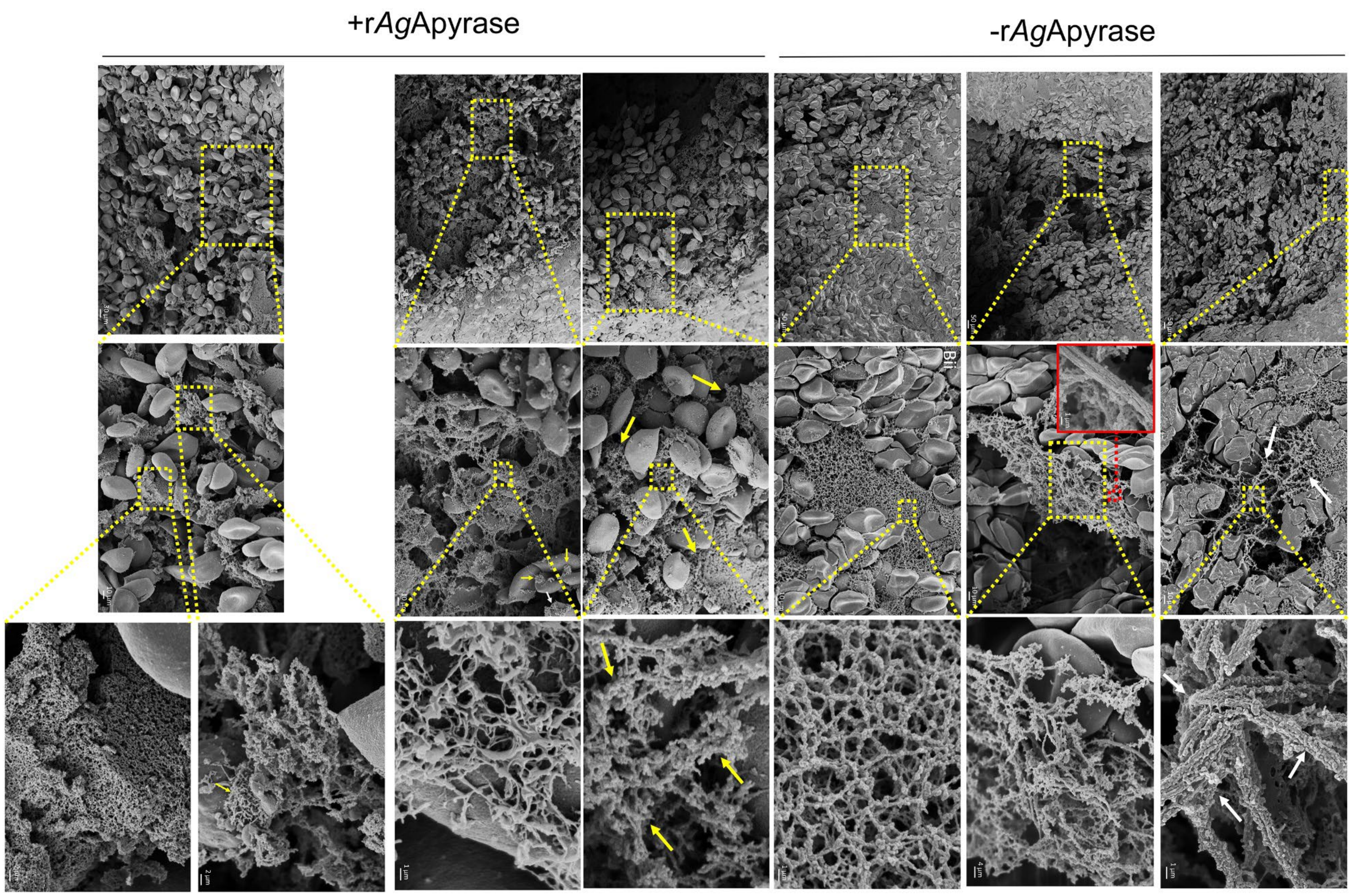

+r*Ag*Apyrase
-r*Ag*Apyrase
Bii

Figure S5: Scanning electron microscopy (SEM) of blood boluses before and after supplementation with rAgApyrase, related to Figures 3 and 4. *An. gambiae* female mosquitoes were fed on mice before or after intravenous injection of rAgApyrase. Midguts were dissected at 30 min post feeding. White arrows indicate aggregated platelets, yellow arrows indicate individual platelets.

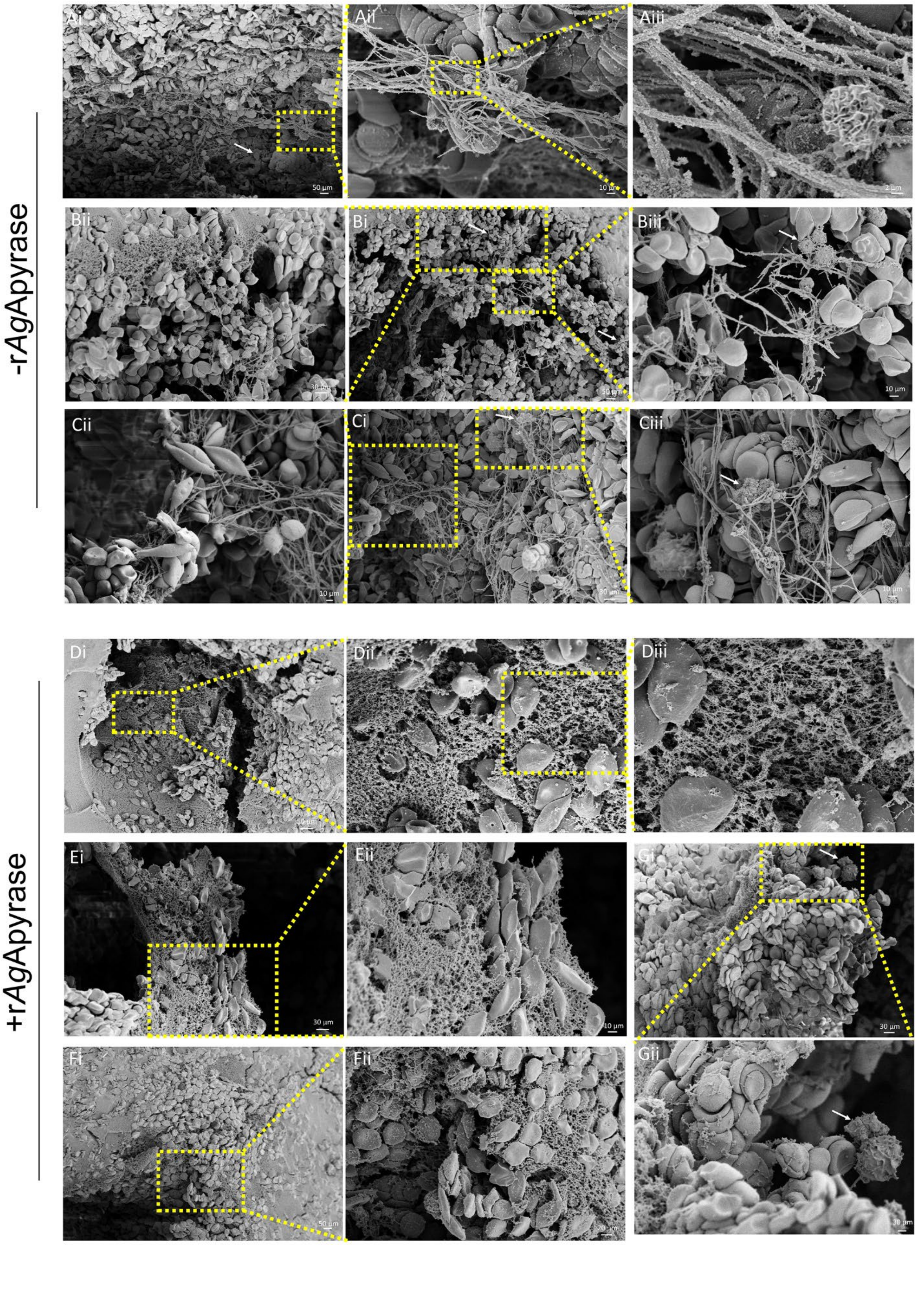
-rAgApyrase
+rAgApyrase
Ai
Aii
Aiii
Bi
Bii
Biii
Ci
Cii
Ciii
Di
Dii
Diii
Ei
Eii
Fi
Fii
Gi
Gii
50 µm
10 µm
2 µm
30 µm
20 µm

Figure S6: Scanning electron microscopy (SEM) of blood boluses before and after supplementation with rAgApyrase, related to Figures 3 and 4. *An. gambiae* female mosquitoes were fed on mice before or after intravenous injection of rAgApyrase. Midguts were dissected at 4 h shown post feeding. White arrows indicate aggregated platelets.

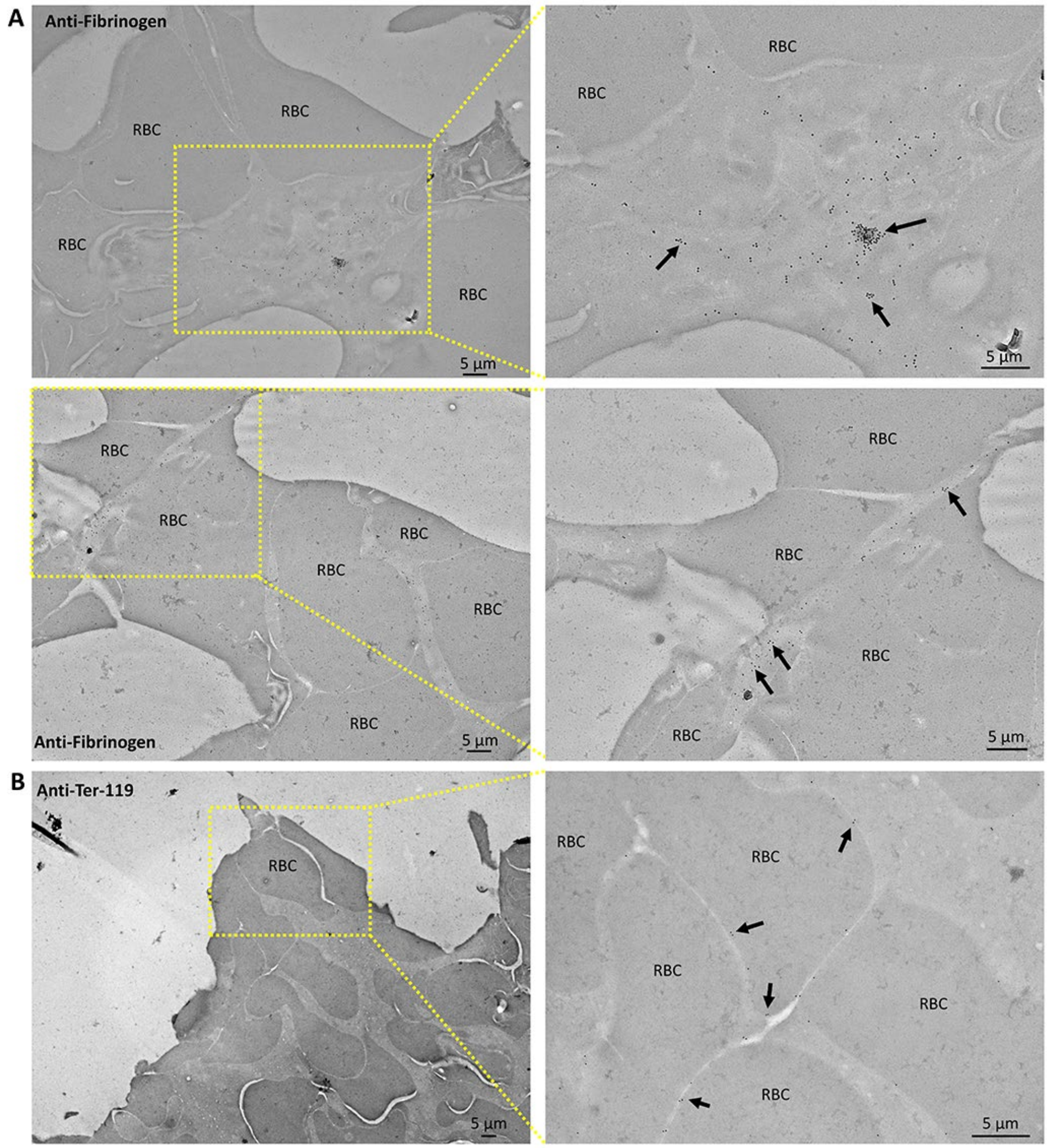

Figure S7: Immuno-TEM detecting fibrinogen in the blood bolus, related to Figure 3. Transmission electron microscopy (TEM) of the blood bolus from mosquito midguts isolated 30 min post blood feeding and stained with anti-fibrinogen or anti-TER-119 antibodies to confirm the presence of fibrinogen/fibrin. In panel (a), the arrows point to the gold particles showing the fibrinogen staining in the regions in between the red blood cells (RBC), whereas in panel (b), the arrows point to the gold particles showing the staining for the RBC surface protein Ter-119.

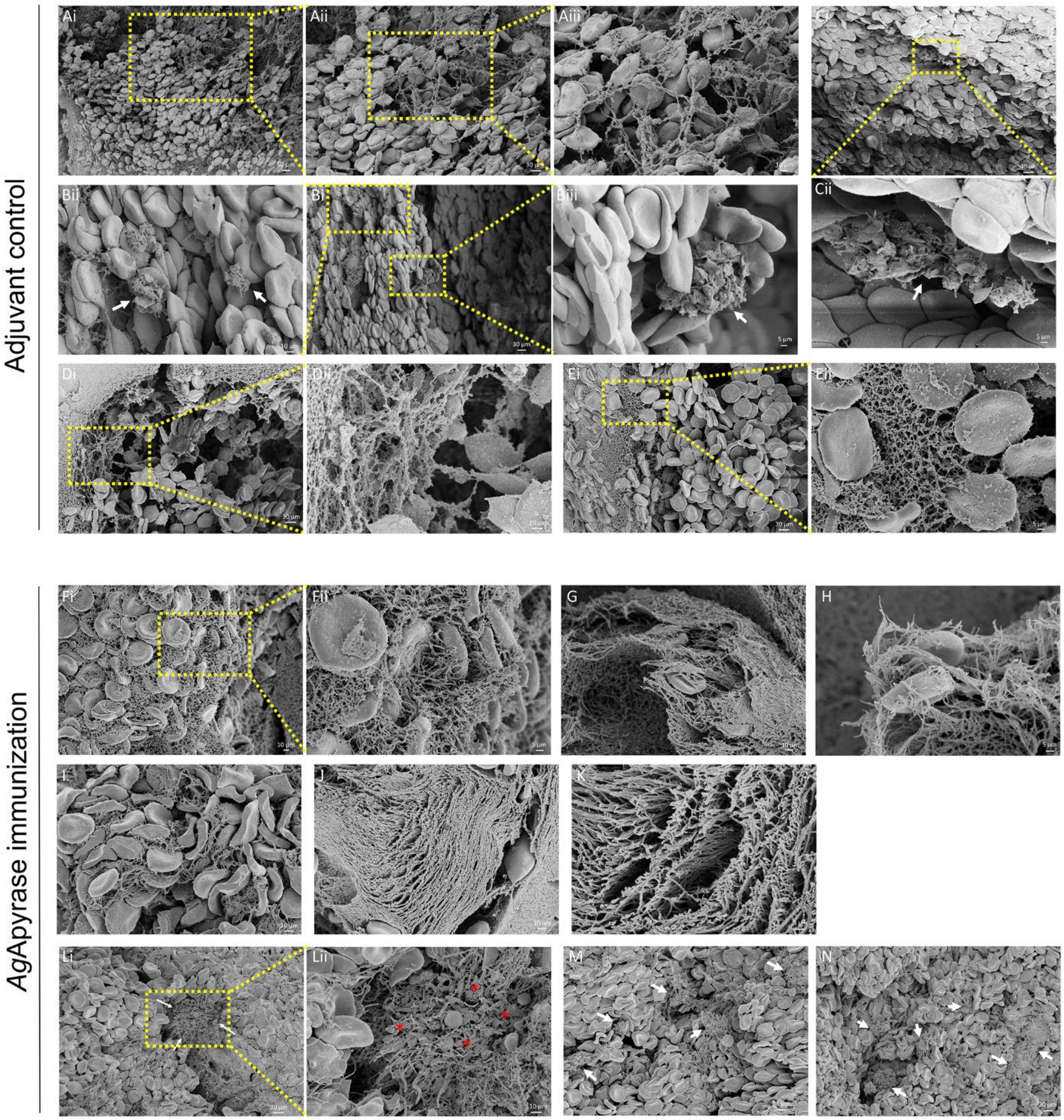

Figure S8: SEM of blood boluses from mosquitoes fed on rAgApyrase immunized mice, related to Figures 3 and 4. Control mosquitoes were fed on mice treated with adjuvant. Midguts were dissected 30 min post feeding. White arrows indicate aggregated platelets. Red asterisks show fragmentation of platelets into smaller vesicular bodies.

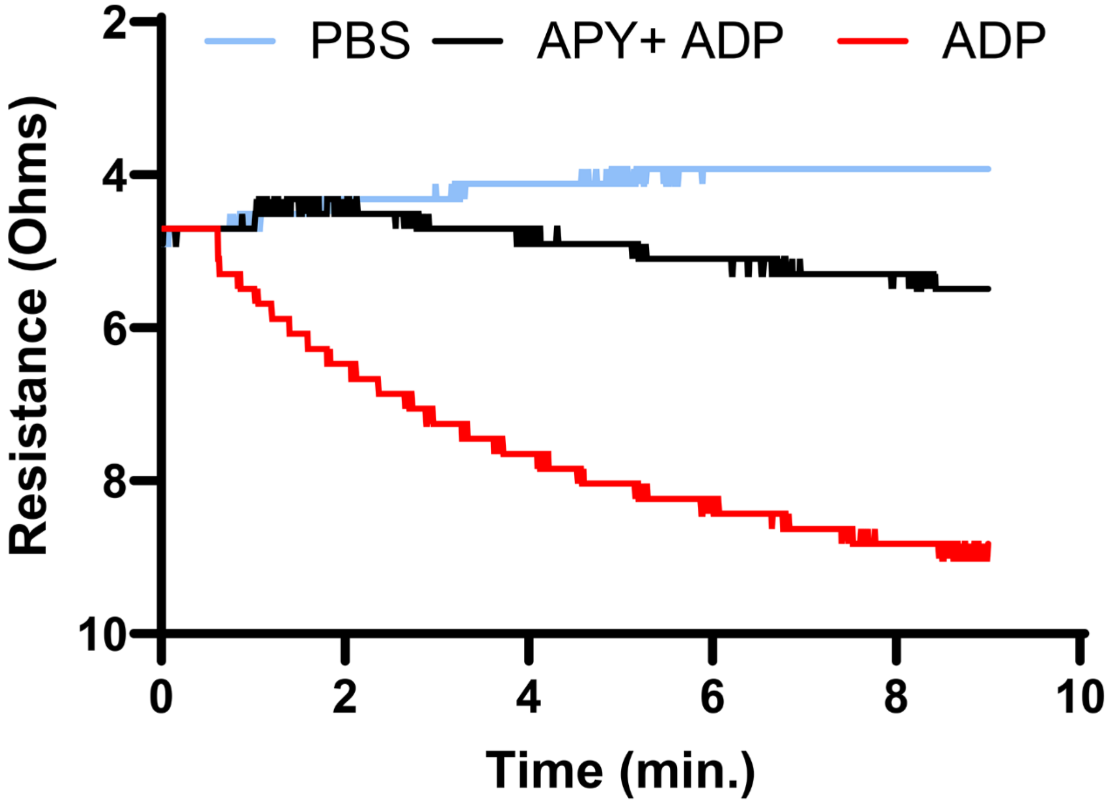

Figure S9: rAgApyrase inhibits the human blood coagulation induced by ADP (red) in contrast to the controls 1X PBS buffer with ADP (blue) & rAgApyrase with ADP which aggregated all the available platelets in human blood, measured by light transmittance.

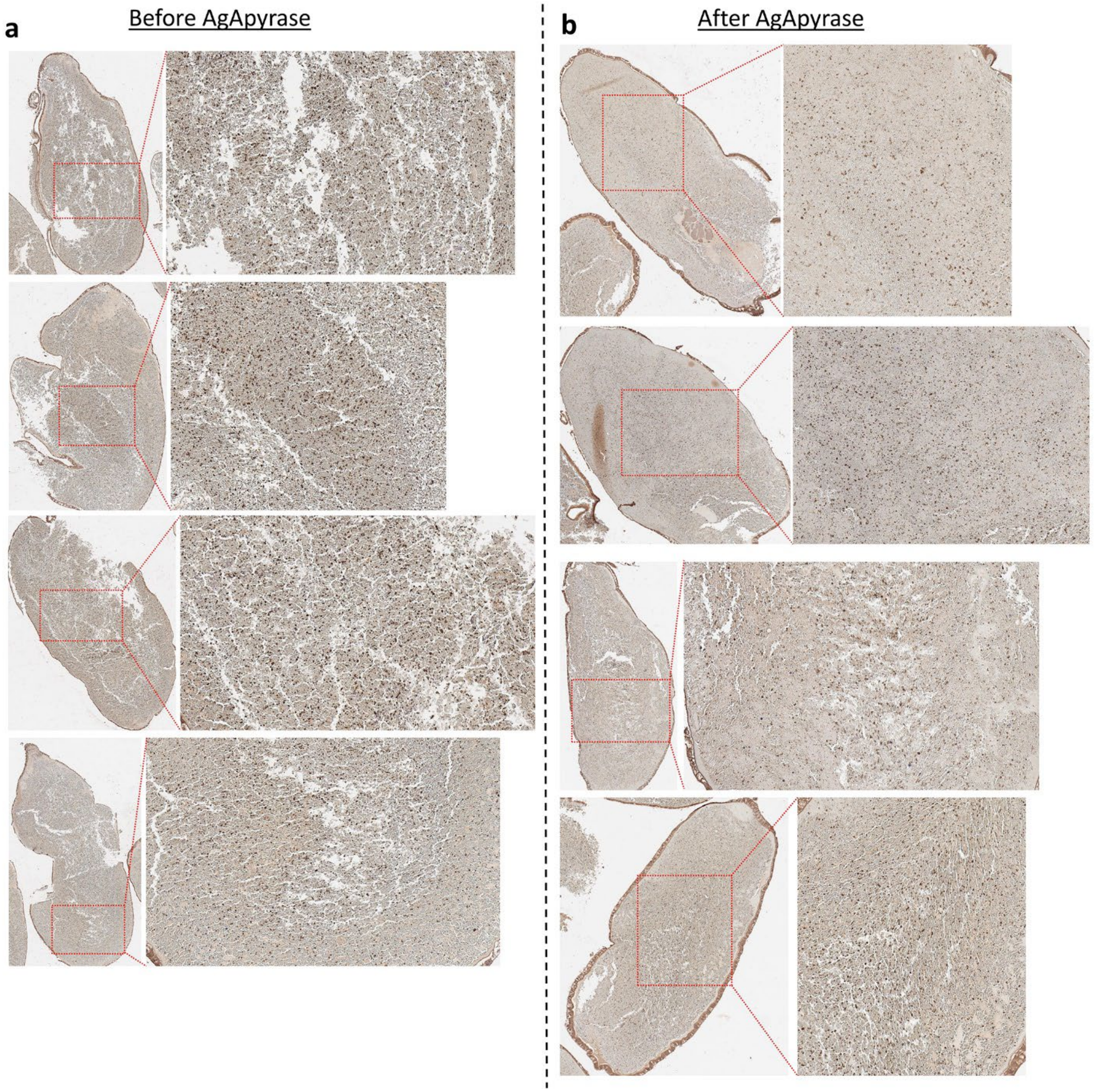

Figure S10: Detection of activated platelets in the mosquito blood bolus, related to Figure 4. Immunohistochemistry of mosquito blood boluses before (a) and after (b) supplementation with rAgApyrase showing platelet activation by staining with P-selectin (dark brown spots). A decrease in staining for the after treatment is observed.

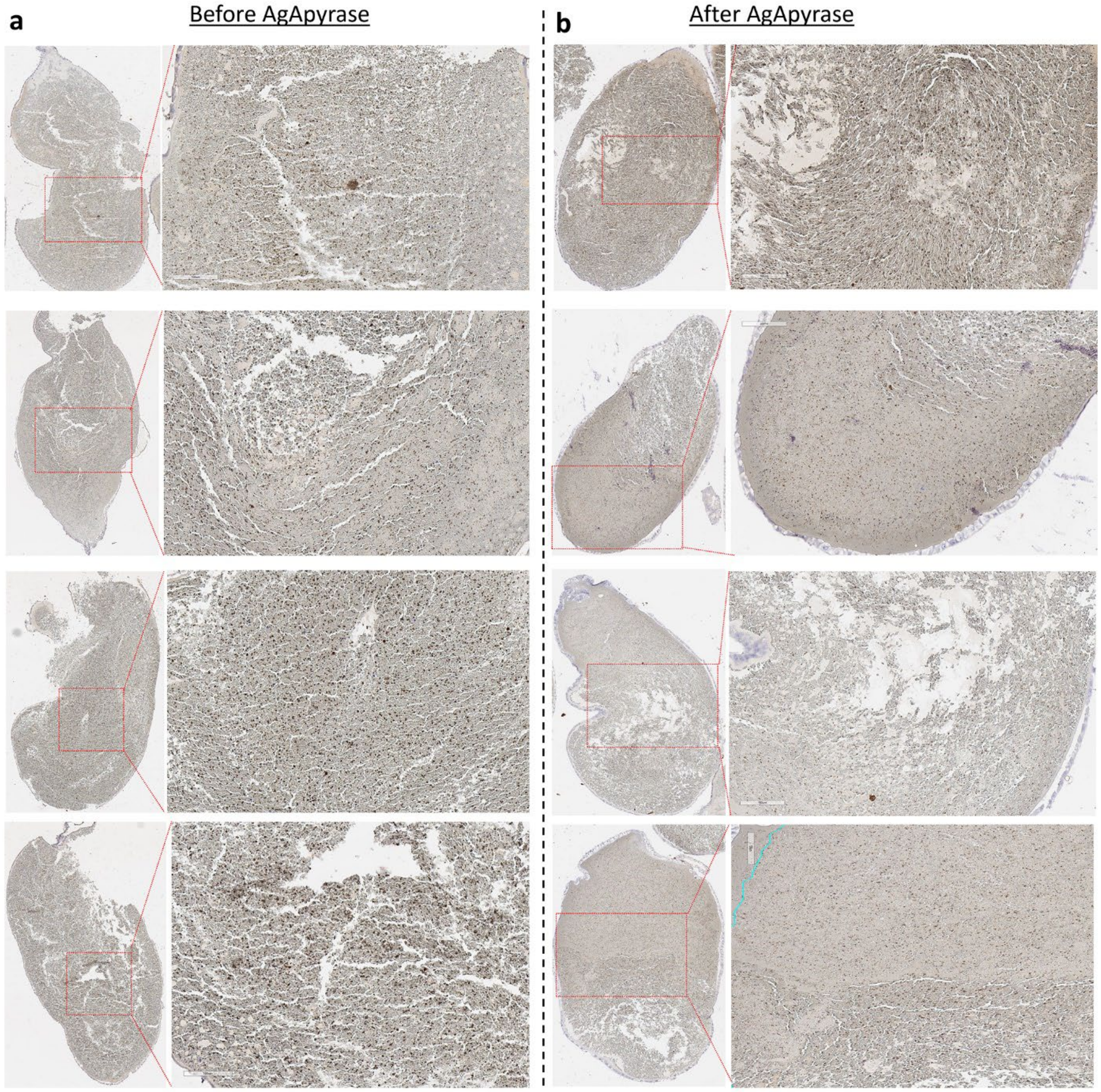

Figure S11: Detection of activated neutrophils in the mosquito blood bolus, related to Figure 5. Immunohistochemistry of mosquito blood boluses before (a) and after (b) supplementation with rAgApyrase stained with an anti-neutrophil elastase antibody (dark brown spots).

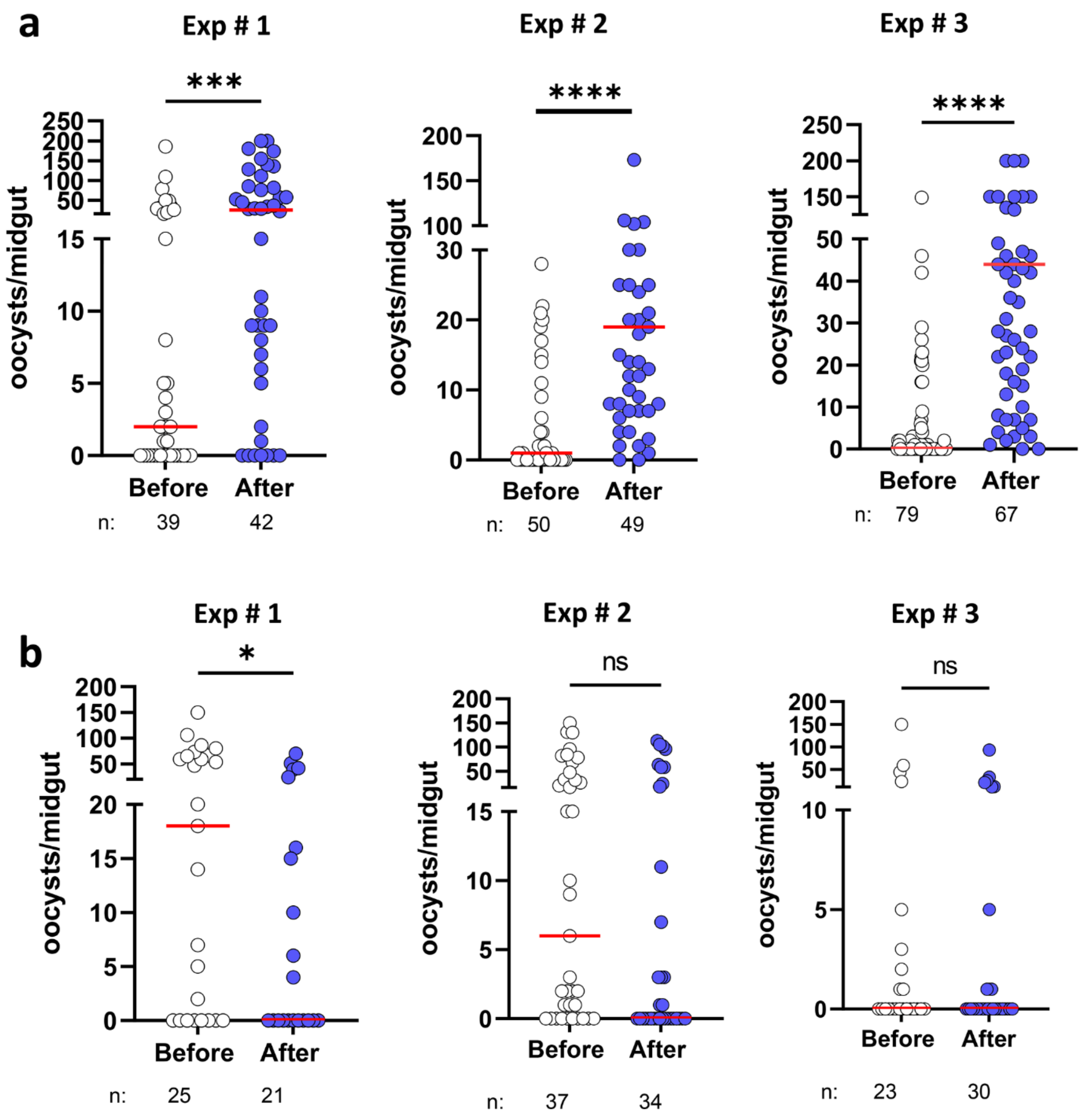

Figure S12: Effect of rAgApyrase on *Plasmodium* infection, related to Figure 6. (**a**) *An. gambiae* mosquitoes were fed on a *P. berghei* infected mouse for 15min (Before group). The same infected mouse was then intravenously supplemented with rAgApyrase. A different group of mosquitoes were fed for 15min (After group) on the rAgApyrase injected mouse. Mosquito midguts were dissected 10 days later, and oocysts were counted. AgApyrase supplementation

increases *Plasmodium* infection of mosquito midguts. Data from three individual experiments shown as pooled data in Figure 6a. (**b**) On supplementation of heat-denatured rAgApyrase, there is a decrease in the *Plasmodium* infection. Data from three individual experiments shown as pooled data in Figure 6a. n= no. of mosquitoes/ experiment. Horizontal red line represents the median oocyst number per mosquito, each dot represents a mosquito. Groups were compared with two-tailed t-test followed by Mann-Whitney comparison test. ****P <0.0001; ***P <0.0006; *P<0.045.

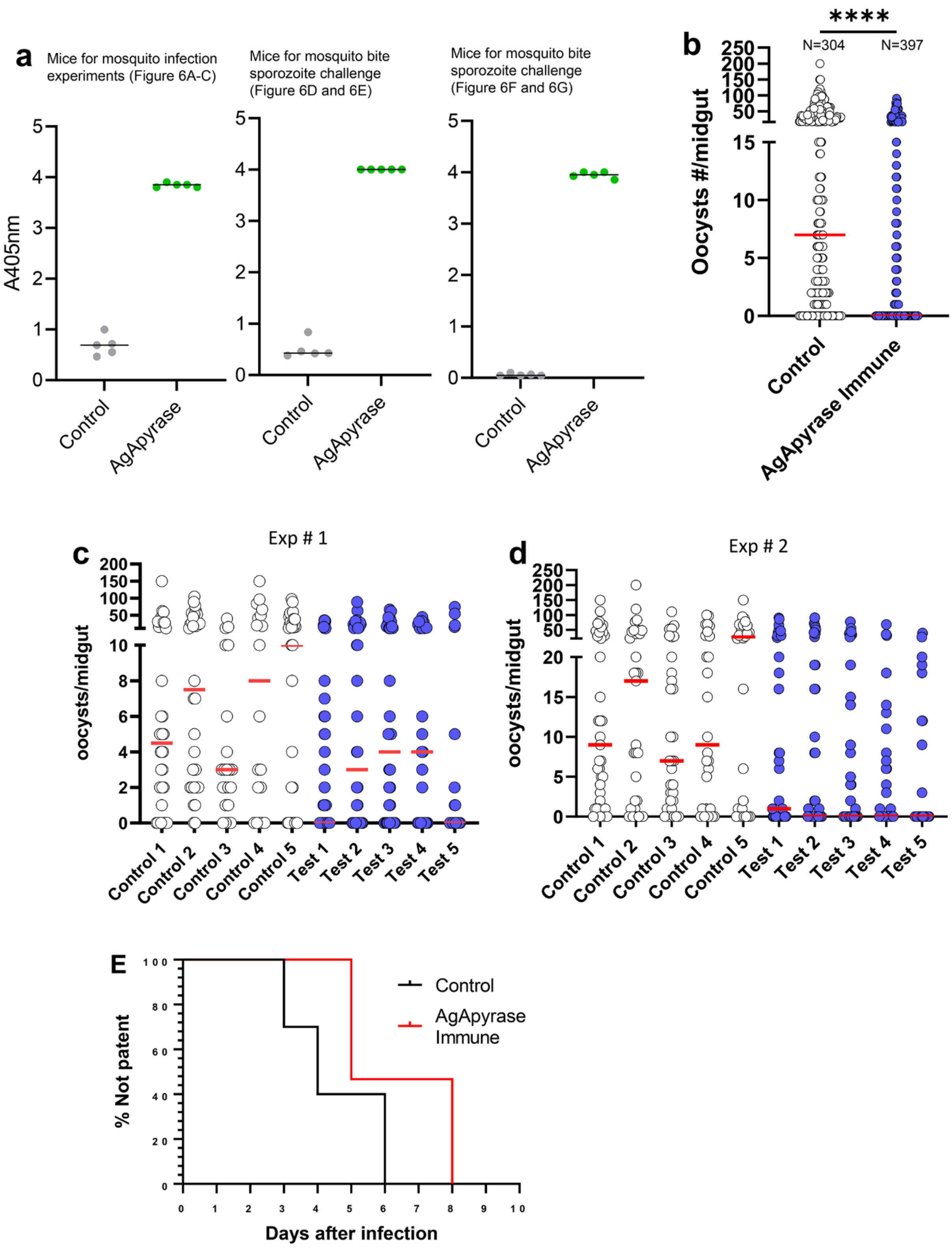
a
Mice for mosquito infection experiments (Figure 6A-C)
Mice for mosquito bite sporozoite challenge (Figure 6D and 6E)
Mice for mosquito bite sporozoite challenge (Figure 6F and 6G)
A405nm
Control
AgApyrase
b
****
N=304
N=397
Oocysts #/midgut
Control
AgApyrase Immune
c
Exp # 1
oocysts/midgut
Control 1
Control 2
Control 3
Control 4
Control 5
Test 1
Test 2
Test 3
Test 4
Test 5
d
Exp # 2
oocysts/midgut
E
% Not patent
Days after infection
Control
AgApyrase Immune

Figure S13. AgApyrase immunization inhibits *Plasmodium* midgut infection, related to Figure 6. (**a**) BALB/c mice were immunized with recombinant AgApyrase using Magic Mouse adjuvant. Adjuvant only was used as a control for the experiment. The antibody titers were determined using ELISA. Each dot represents data from one animal. (**b**) The control and immunized mice were infected with *P. berghei* and *An. gambiae* mosquitoes were fed for 15min. Mosquito midguts were dissected 10 days later, and oocysts were counted. AgApyrase significantly inhibits *Plasmodium* midgut infection. Pooled data for no. of oocysts/ midgut fed on 10 individual animals shown. (**c and d**) Data from mosquitoes fed on each individual animal of the data shown in panel (b). Data obtained from two independent immunization experiments (experiment #1 and #2). Control: Adjuvant only; Test: Apyrase Immunized. n= no. of mosquitoes. (**e**) Pre-patency for control and apyrase immunized mice. Sporozoite infectivity was determined by the day of appearance of blood-stage parasites in peripheral blood (patency) by Giemsa staining. Data pooled from two independent experiments shown in Figure 6e.
