# Supplementary material for: Mosquito salivary apyrase regulates blood meal hemostasis and facilitates malaria parasite transmission
Source: Nat Commun. 2024 Sep 18;15:8194. doi: 10.1038/s41467-024-52502-3 (PMC11410810; doi:10.1038/s41467-024-52502-3)
Supplement: Supplementary file 3 — Description of Additional Supplementary Files [file 41467_2024_52502_MOESM3_ESM.pdf]

# Description of Additional Supplementary Files

**Supplementary Data 1:** Proteins detected by mass spectrometry analysisi of fractions Z7, Z8, Z9, A5 and B3, Related to Figure 1. Salivary gland extracts were subjected to size-exclusion chromatography and each fraction was tested in the sc-tPA activity assay. Fractions showing activity (Z7-9) along with two different fractions showing no activity (A5 and B3) were analyzed by mass spectrometry.

**Supplementary Data 2:** Proteins shortlisted as tPA activator candidates, Related to Figure 1. Proteins identified by mass spectrometry were shortlisted based on the presence of secretion signal, presence in fractions Z7-9, and absence from fractions A5 and B3 which did not activate tPA.
