# Supplementary material for: Mosquito salivary apyrase regulates blood meal hemostasis and facilitates malaria parasite transmission
Source: Nat Commun. 2024 Sep 18;15:8194. doi: 10.1038/s41467-024-52502-3 (PMC11410810; doi:10.1038/s41467-024-52502-3)
Supplement: Supplementary file 6 — Reporting Summary [file 41467_2024_52502_MOESM6_ESM.pdf]

nature portfolio

Corresponding author(s): Joel Vega-Rodriguez
Last updated by author(s): 5/9/2024

Reporting Summary

Nature Portfolio wishes to improve the reproducibility of the work that we publish. This form provides structure for consistency and transparency in reporting. For further information on Nature Portfolio policies, see our Editorial Policies and the Editorial Policy Checklist.

Statistics

For all statistical analyses, confirm that the following items are present in the figure legend, table legend, main text, or Methods section.

n/a	Confirmed	
☐	☒	The exact sample size (*n*) for each experimental group/condition, given as a discrete number and unit of measurement
☐	☒	A statement on whether measurements were taken from distinct samples or whether the same sample was measured repeatedly
☐	☒	The statistical test(s) used AND whether they are one- or two-sided<br>*Only common tests should be described solely by name; describe more complex techniques in the Methods section.*
☐	☒	A description of all covariates tested
☐	☒	A description of any assumptions or corrections, such as tests of normality and adjustment for multiple comparisons
☐	☒	A full description of the statistical parameters including central tendency (e.g. means) or other basic estimates (e.g. regression coefficient) AND variation (e.g. standard deviation) or associated estimates of uncertainty (e.g. confidence intervals)
☐	☒	For null hypothesis testing, the test statistic (e.g. *F*, *t*, *r*) with confidence intervals, effect sizes, degrees of freedom and *P* value noted<br>*Give P values as exact values whenever suitable.*
☒	☐	For Bayesian analysis, information on the choice of priors and Markov chain Monte Carlo settings
☒	☐	For hierarchical and complex designs, identification of the appropriate level for tests and full reporting of outcomes
☒	☐	Estimates of effect sizes (e.g. Cohen's *d*, Pearson's *r*), indicating how they were calculated

Our web collection on statistics for biologists contains articles on many of the points above.

Software and code

Policy information about availability of computer code

Data collection	Electron microscopy images were collected on a Hitachi SU8000 field emission scanning electron microscope (Hitachi, High-Tech in America).<br>Spectophotometry data were collected on a Cytation 5 ® Cell Imaging Multi-Mode Reader (BioTek Instruments).<br>Histology images were collected on an AOlympus BX51 light microscope using an Olympus DP73 camera.<br>Western blot data was collected on an iBright imager (ThermoFisher).<br>Platelet aggregation data was collected using a Chrono-Log aggregometer model 700 (Chrono-Log Corporation).<br>Mouse whole body luciferase signa was recorded using an IVIS Spectrum imager (PerkinElmer)
Data analysis	Microscopy image processing was performed using Imaris 9.2.1 (Bitplane).<br>Data for spectophotometry assays were analyzed with the Gen5 Image Prime software version 3.04. (BioTek Instruments)<br>Western blot data was analyzed using the Gen5 Image Prime software version 3.04. (BioTek Instruments)<br>Sequences were aligned using Clustal Omega as implemented in JalView version 2.11.2.6.<br>Statistical analyses were done using Graphpad Prism version 9.0

For manuscripts utilizing custom algorithms or software that are central to the research but not yet described in published literature, software must be made available to editors and reviewers. We strongly encourage code deposition in a community repository (e.g. GitHub). See the Nature Portfolio guidelines for submitting code & software for further information.

Data

Policy information about availability of data

All manuscripts must include a data availability statement. This statement should provide the following information, where applicable:

- Accession codes, unique identifiers, or web links for publicly available datasets
- A description of any restrictions on data availability
- For clinical datasets or third party data, please ensure that the statement adheres to our policy

The datasets generated during and/or analysed during the current study are available from the corresponding author on reasonable request.

Research involving human participants, their data, or biological material

Policy information about studies with human participants or human data. See also policy information about sex, gender (identity/presentation), and sexual orientation and race, ethnicity and racism.

Reporting on sex and gender	Platelets were collected under NIH protocol 99-CC-0168 "Collection and Distribution of Blood Components from Healthy Donors for In Vitro Use"<br>Gender: Male & Female
Reporting on race, ethnicity, or other socially relevant groupings	Platelets were collected under NIH protocol 99-CC-0168 "Collection and Distribution of Blood Components from Healthy Donors for In Vitro Use"<br>Gender: Male & Female
Population characteristics	Recruitment:<br>Type: Participants currently recruited/enrolled<br>Gender: Male & Female<br>Min Age: 18 Years<br>Max Age: N/A<br><br>Exclusion: Children;<br>Adults who are or may become unable to consent;<br>Neonates;<br>Pregnant Women
Recruitment	Recruitment:<br>Type: Participants currently recruited/enrolled<br>Gender: Male & Female<br>Min Age: 18 Years<br>Max Age: N/A<br><br>Exclusion: Children;<br>Adults who are or may become unable to consent;<br>Neonates;<br>Pregnant Women
Ethics oversight	National Institutes of health

Note that full information on the approval of the study protocol must also be provided in the manuscript.

Field-specific reporting

Please select the one below that is the best fit for your research. If you are not sure, read the appropriate sections before making your selection.

☒ Life sciences ☐ Behavioural & social sciences ☐ Ecological, evolutionary & environmental sciences

For a reference copy of the document with all sections, see nature.com/documents/nr-reporting-summary-flat.pdf

Life sciences study design

All studies must disclose on these points even when the disclosure is negative.

Sample size	No statistical methods were used to determine sample size. Sample size was determined to be adequate based on experimental consistency and effect, and previous publications. Generally three independent biological replicates were done for each experiment.
Data exclusions	No data were excluded from analysis

Replication	All replicates were successful and included in data analysis
Randomization	Randomization was not necessary for our experiments.
Blinding	Blinding wasn't necessary

Reporting for specific materials, systems and methods

We require information from authors about some types of materials, experimental systems and methods used in many studies. Here, indicate whether each material, system or method listed is relevant to your study. If you are not sure if a list item applies to your research, read the appropriate section before selecting a response.

Materials & experimental systems

n/a	Involved in the study
☐	☒ Antibodies
☐	☒ Eukaryotic cell lines
☒	☐ Palaeontology and archaeology
☐	☒ Animals and other organisms
☒	☐ Clinical data
☒	☐ Dual use research of concern
☒	☐ Plants

Methods

n/a	Involved in the study
☒	☐ ChIP-seq
☒	☐ Flow cytometry
☒	☐ MRI-based neuroimaging

Antibodies

Antibodies used	Anti-sc-tPA (Innovative Research, Novi, MI, USA, #ISHAHUTPAAP100UG)<br>Donkey anti-sheep IgG labeled with horse radish peroxidase (Abcam, Waltham, MA, USA, #ab6900)<br>mouse Anti-AgApyrase (made in house at Vega-Rodriguez Lab, NIH)<br>rabbit anti-tPA (Millipore-Sigma, Marlborough, MA, USA)<br>rabbit polyclonal CD62p (Bioss, Woburn, MA, USA, #Bs-0561R)<br>anti-fibrinogen (Millipore--Sigma, Marlborough, MA, USA #341552)<br>anti-TER-119 (BD Biosciences, San Jose, CA, USA #550565)<br>anti-MPO (Dako)
Validation	Anti-sc-tPA (Innovative Research, Novi, MI, USA, #ISHAHUTPAAP100UG)<br>Donkey anti-sheep IgG labeled with horse radish peroxidase (Abcam, Waltham, MA, USA, #ab6900)<br>mouse Anti-AgApyrase (made in house at Vega-Rodriguez Lab, NIH)<br>rabbit anti-tPA (Millipore-Sigma, Marlborough, MA, USA)<br>rabbit polyclonal CD62p (Bioss, Woburn, MA, USA, #Bs-0561R)<br>anti-fibrinogen (Millipore--Sigma, Marlborough, MA, USA #341552)<br>anti-TER-119 (BD Biosciences, San Jose, CA, USA #550565)<br>anti-MPO (Dako)

Eukaryotic cell lines

Policy information about cell lines and Sex and Gender in Research

Cell line source(s)	FreeStyle 293F cells (Thermo Fisher Scientific, Waltham, MA, USA, #R79007)
Authentication	FreeStyle 293F cells (Thermo Fisher Scientific, Waltham, MA, USA, #R79007)
Mycoplasma contamination	Company test for Mycoplasma contamination - FreeStyle 293F cells (Thermo Fisher Scientific, Waltham, MA, USA, #R79007)
Commonly misidentified lines (See ICLAC register)	*Name any commonly misidentified cell lines used in the study and provide a rationale for their use.*

Animals and other research organisms

Policy information about studies involving animals; ARRIVE guidelines recommended for reporting animal research, and Sex and Gender in Research

Laboratory animals	Anopheles stephensi, Liston strain (Feldmann et al., 1989), Female, 3-21 days old<br>Swiss webster or BALB/c mice, Female, 4-6 weeks old, NIAID animal study proposals LMVR-22
Wild animals	The study did not involve wild animals

Reporting on sex	NA
Field-collected samples	The study did not involve wild animals
Ethics oversight	Studies with animals were approved by the Institutional Animal Care and Use Committee (IACUC), Care and Use Committees (NIAID ACUC)<br>Swiss webster mice, Female, 4-6 weeks old, NIAID animal study proposals LMVR-22

Note that full information on the approval of the study protocol must also be provided in the manuscript.
